# Uncertainty related to multigene panel testing for cancer: a qualitative study on counsellors’ and counselees’ views

**DOI:** 10.1007/s12687-018-0393-1

**Published:** 2018-11-14

**Authors:** Niki M. Medendorp, Marij A. Hillen, Laxsini Murugesu, Cora M. Aalfs, Anne M. Stiggelbout, Ellen M. A. Smets

**Affiliations:** 1 Department of Medical Psychology – Amsterdam UMC, University of Amsterdam, P.O. Box 22700, 1100 DE Amsterdam, The Netherlands; 2 Amsterdam Public Health research institute, Amsterdam, The Netherlands; 3 Cancer Center Amsterdam, Amsterdam, The Netherlands; 4 Department of Clinical Genetics – Amsterdam UMC, University of Amsterdam, Amsterdam, The Netherlands; 5 Department of Clinical Genetics – Amsterdam UMC, Vrije Universiteit Amsterdam, Amsterdam, The Netherlands; 6 Medical Decision Making, Department of Biomedical Data Sciences, Leiden University Medical Center, Leiden, The Netherlands

**Keywords:** Multigene panel testing, Uncertainty, Genetic counselling, Experiences, Focus groups, Interviews

## Abstract

Multigene panel testing is mainly used to improve identification of genetic causes in families with characteristics fitting multiple possible cancer syndromes. This technique may yield uncertainty, for example when variants of unknown significance are identified. This study explores counsellors’ and counselees’ experiences with uncertainty, and how they discuss uncertainties and decide about multigene panel testing. Six focus groups were conducted including 38 counsellors. Twelve counselees who had received genetic counselling about a multigene panel test were interviewed. The focus group sessions and interviews were audio-recorded and transcribed verbatim. Transcripts were analysed inductively by two independent coders and data were examined to obtain a comprehensive list of themes. Counsellors identified several uncertainties, e.g. finding a variant of unknown significance, or detecting an unsolicited finding. Most difficulty was experienced in deciding what uncertain information to communicate to counselees and how to do so. The extent and manner of providing uncertain information differed between centres and between counsellors. Counsellors attached more value to counselees’ preferences in decision making compared to less extended tests. Counselees experienced difficulty in recalling which uncertainties had been discussed during genetic counselling. They primarily reported to have experienced uncertainty about their own and their relatives’ risk of developing cancer. Counselees felt they had had a say in the decision. This study showed that counsellors need more guidance on whether and how to convey uncertainty. Undesirable practice variation in the communication of uncertainty may be prevented by determining what information should minimally be discussed to enable informed decision making.

## Introduction

Next-generation sequencing (NGS) technologies are the result of continuous developments in the field of clinical genetics. NGS techniques are capable of sequencing multiple genes at one time to identify genetic predispositions (Hall et al. 2014). In a diagnostic setting, NGS is mainly used in the form of multigene panels (Biesecker and Green 2014; Mardis 2011; Weiss et al. 2013). Gene panels for cancer include various genes associated with cancer, varying in the number of included genes between genetic centres (Xue et al. 2014). Generally, both genes associated with high and modest risks for cancer can be sequenced (Stratton and Rahman 2008), despite the possibility of scientific uncertainty about their clinical value (Domchek et al. 2013; Hall et al. 2014; Reis-Filho 2009). In the Netherlands, multigene panels are mainly offered when an indication exists for multiple hereditary tumour syndrome testing, based on family history (i.e. families with characteristics fitting multiple possible genetic syndromes) (Stichting Opsporing Erfelijke Tumoren (STOET) and Vereniging Klinische Genetica Nederland (VKGN) 2017), or when targeted tests generate uninformative test results and follow-up diagnostics are desired because a hereditary cause is still suspected (Hall et al. 2014; Kurian and Ford 2015). In most cases, based on family history, counsellors offer one panel to counselees, or, in some cases, various panels when multiple are applicable for the counselee’s situation (Hall et al. 2014).

Although multigene panel testing may be useful for identifying a hereditary cause for cancer, it can complicate the counselling process as it may involve uncertainties; first for researchers and counsellors (Howard and Iwarsson 2017). Performing multigene panel tests increases the possibility of yielding uncertain test results compared to targeted tests (Bradbury et al. 2015). For example, there is an increased chance of identifying a variant of which the meaning and implications are unknown, the so-called ‘variants of unknown significance’ (VUS) (Domchek et al. 2013; Hall et al. 2014). VUS may burden counsellors with uncertainty about what action to undertake, for example, whether or not to offer screening, and may consequently lead to possible dispensable screening recommendations (Hall et al. 2014). Also, multigene panel tests increase the possibility of generating findings unrelated to phenotypical characteristics in the patient or family, i.e. unsolicited findings. These findings occur when genes are tested that are associated with the specific cancer type(s) present in the counselee/family, and/or with other cancer types or diseases, for many of which testing would not otherwise be considered (Hall et al. 2014). Uncertain test results may bring up uncertainty about their clinical utility, i.e. how useful it is for a patient to obtain the (genetic) information, and is therefore debated among counsellors (Howard and Iwarsson 2017).

During both pre-test and post-test genetic counselling, counsellors need to deal with the above-mentioned uncertainties; for example they need to decide *whether*, *to what extent* and *how* to discuss uncertainty with counselees (Portnoy et al. 2013). A study of Blazer et al. on perceptions, experiences and needs among clinicians (including genetic counsellors) showed that clinicians reported low self-confidence in interpreting and counselling about multigene panels and had concerns about lack of evidence to inform clinical utility (Blazer et al. 2015). Moreover, a recent study on the experiences of clinicians and patients with VUS (Clift et al. 2018) reported that clinicians expressed doubts about returning VUS, and urged on returning them with caution and trying to avoid unrealistic hope about having found the cause for the disease among patients. Patients on the other hand reported they wanted to receive information, despite uncertainty (Clift et al. 2018). How much to discuss during *pre-test* counselling is also unclear. Some studies argue that, despite the possible harmful consequences, possible uncertainties should be discussed before panel testing, to facilitate informed decision making (Bradbury et al. 2015; Fecteau et al. 2014). Although several studies have focused on discussing medical uncertainty, to our knowledge, no empirical evidence is available on how counsellors deal with uncertainty about multigene panels during genetic counselling, and how difficult it is for them to discuss uncertainty and involve patients in the decision about whether or not to perform multigene panel testing.

Moreover, multigene panels may evoke uncertainty not only among counsellors but also for counselees (Han et al. 2017; Lumish et al. 2017). For example, during pre-counselling, their uncertainty may relate to the probability of carrying a mutation or develop cancer. During post-test counselling, counselees may experience uncertainty regarding their future when the consequences of test results are unknown (Johnston et al. 2012; Niemitz 2007). There have been several studies on the effects of uncertain test results. For example, Lumish et al. investigated the impact of multigene panel testing and showed that patients with a positive or VUS test result had the highest scores for intrusive thoughts, avoidance, and distress (Lumish et al. 2017). However, to our knowledge, no research has been done on the experiences with uncertainty of counselees, and it is therefore unknown whether and how counselees experience uncertainties during pre- and post-test counselling about a multigene panel test, and whether and to what extent their experiences relate to those of their counsellors.

Therefore, this study aimed to i) gain insight into counsellors’ and counselees’ experiences with uncertainty regarding multigene panel testing, and ii) their views on discussing uncertainty and making decisions about multigene panel testing during cancer genetic counselling.

## Methods

### Study design

This qualitative study used focus groups and personal interviews to explore how uncertainty is experienced and discussed, and how decisions regarding multigene panel testing are generally made. More specific, this study focused on gene panels that also include genes having questionable associations with cancer risks. Focus groups enable the exploration of the variety in opinions and motivations, by using interaction and discussion between participants (Basch 1987; Kitzinger 1994). Initially, it was planned to organise separate focus groups for counsellors and for counselees. However, the relatively long time needed to recruit sufficient counselees for a focus group session (because discussions on multigene panels were less prevalent than expected) would make the period between genetic counselling and the focus group too long. To avoid possible hindering of recall of the counselling session due to this long period, we chose to interview counselees individually shortly after their counselling session.

The Medical Ethics Review Board of the Academic Medical Center approved the study protocol. No ethico-legal adjudication was required as this study had no serious impact on the participants and did not interfere with standard care.

### Recruitment and sample

For this study, two groups were recruited: counsellors (i.e. clinical geneticists, interns and residents, and genetic counsellors) and counselees (i.e. patients with cancer, or relatives of someone with cancer, who had sought genetic services).

Counsellors from any of the eight centres in the Netherlands where genetic testing is performed and who are involved in cancer genetic counselling were eligible for participation. In each centre, a contact person provided details of interested counsellors to the primary researcher (NM) who subsequently provided these persons with more information by telephone, and sent them an information letter. After consent was received, a focus group session was planned in consultation with a local research coordinator.

Counselees were eligible when they 1) were aged ≥ 18 years, 2) spoke adequate Dutch, 3) visited one of the eight Dutch genetic centres and 4) had discussed a multigene panel test during cancer genetic counselling in the previous 4 months. Eligible counselees were informed about the study by their counsellor at the end of their consultation, or by a research coordinator by telephone. They received an information letter and were asked permission to be contacted by the researcher (NM). The researcher gave them more details about the study by telephone. Interested counselees were then sent an information letter and a consent form by mail. After return of the signed informed consent form, an appointment for the interview was planned.

### Data collection

#### Counsellors

Focus group sessions took place in the participating centres between September 2016 and January 2017; each session lasted 1.5–2 h. Upon arrival, counsellors filled out a questionnaire assessing their socio-demographic characteristics (i.e. age, gender, profession and years of experience). Next, one moderator (NM) and one observer (LM), with a background in psychology and health sciences, respectively, guided the sessions using a topic list developed in consultation with the research team (see Appendix Table 2). The focus group started with an explanation of the concerning gene panels and the notification that all uncertainties related to these panels are topic of interest. Subsequently, the moderator presented various predefined questions; counsellors were invited to respond to these questions and/or to the responses from their colleagues. The observer took notes to enable clarification of the audio-recordings and to preserve subtle differences in the responses and statements of the counsellors during analysis.

#### Counselees

All interviews with counselees were conducted between January and April 2017 by one of two researchers (NM and LM) using an interview guide (see Appendix Table 3). Depending on the counselee’s preference, interviews were conducted at their home or in a quiet place elsewhere; each interview lasted ± 45 min. Prior to the start of the interview, counselees filled out a questionnaire addressing their socio-demographic (i.e. age, gender and educational level) and medical characteristics including the reason for visiting, the type and number of familial cancer(s), the initiator of the referral for genetic counselling, and whether a multigene panel test was ordered afterwards.

### Data analysis

Focus groups and interviews were audio-recorded and transcribed verbatim by two researchers (NM and LM). Initially, the datasets of the focus group sessions and interviews were analysed separately. First, salient text fragments were provided with initial codes (open coding) (Murray and Chamberlain 1999) by NM and LM (both experienced in coding and thematically analysing qualitative data) independently, facilitated by MAXQDA software for qualitative research (MAXQDA 2004). During weekly meetings, NM and LM discussed detailed codes to reach consensus. The developed coding schemes were then discussed with a third researcher (MH), once during coding and again after completing the coding. Next, codes of the focus groups and interviews were collated into separate categories. Observations made during the focus groups were used to contextualise the analysis of the focus groups. Then, both sets of categories were examined to identify convergences and divergences between the groups. During this process, a comprehensive list of themes was compiled to reflect the separate categories. Finally, these themes were critically reviewed by all members of the project team.

## Results

### Sample characteristics

All counsellors asked for participation agreed, resulting in a total of 38 counsellors from six of the eight Dutch genetic centres; each focus group included 4–10 counsellors. In addition, 12 counselees agreed with participation and were individually interviewed. Table 1 lists the characteristics of all 50 participants. Of the counselees who received their test result prior to the interview no one received an uncertain test result.

**Table 1:** Characteristics of the two groups of study participants

	*n* (%)
Counsellors (*n* = 38)	
Mean age in years ± SD (range)	43 ± 10.0 (26–62)
Male	4 (10.5)
Professional training	
Clinical geneticist	19 (50)
Genetic counsellor	13 (34.2)
Intern	3 (7.9)
Resident	3 (7.9)
Mean work experience in years ± SD (range)	10.5 ± 6.1 (0–25)
Counselees (*n* = 12)	
Mean age in years ± SD (range)	54 ± 14.4 (28–73)
Male	6 (50)
Educational level	
Low: none/primary school	2 (16.7)
Intermediate: secondary/intermediate voc. education	2 (16.7)
High: higher education/university	8 (66.7)
Cancer patient	9 (75)
Relative of cancer patient	5 (41.7)
Test results received at time of interview	
Yes	5 (41.7)
Not yet	7 (58.3)

### Experiences with uncertainty

Counsellors confirmed that multigene panel testing involves many uncertainties. Moreover, they reported that uncertainties tend to multiply, since this technique involves testing an increasing number of genes. For that reason, some expressed a need for less extended panels:

If 36 genes are tested, genes that are less known are also included. Therefore, uncertainty increases, for example because the chance of finding an uncertain variant increases.

Some panels are too large. I really want separate gene panels to reduce their size and, therefore, reduce the chance of finding something unknown. (FG 002)

Counsellors had various uncertainties related to multigene panel tests, both before and after testing. For example, before testing, counsellors experienced uncertainty about deciding *when*, *in which* counselee or family, and *which* panel test should be performed:

The uncertainty ... do we need to carry out a panel test or not, what does it add? That’s what we discuss among counsellors here. Shall we perform it and in which persons? (FG 005)

Counsellors also experienced uncertainty about the amount of uncertain information they should discuss before testing:

I find it difficult to determine the amount of information I need to provide. On the one hand it can be enough to say we have a panel test including seven causes of breast cancer [….], but I have the feeling that I’ve only explained the tip of an iceberg. (FG 002)

Nevertheless, counsellors found the post-test counselling more difficult than the pre-test counselling due to uncertainties emerging from an increased number of (less well-known) genes included in multigene panel tests, e.g. due to i) VUS and their questionable clinical utility, ii) unsolicited findings and unknown unsolicited findings (i.e. findings not related to the reason for testing, although they are/might be of clinical value), and iii) the imperfect sensitivity for detecting a genetic predisposition (i.e. not detecting a mutation does not necessarily imply that the counselee does not carry one):

(1) I think pre-test counselling is not really that difficult. People have a choice, we talk about it and I actually really like it. It’s more difficult to counsel when a VUS in a BRCA2 has been found … what should we do about the sister? I find that more difficult than discussing a panel test beforehand. (FG 006)

(2) On a more technical level there are several uncertainties related to the test result: unknown variant in known genes, unknown variant in unknown genes, known variants in unknown genes and so on. I find it difficult because occasionally we pick up one of those uncertain variants.

If the clinical picture is still insufficient, we know nothing about the risks, screening, penetrance, clinical utility for this patient. (FG 001)

Counsellors experienced three forms of uncertainty during post-test counselling related to uncertain test results. First, regarding what to communicate, and how to inform counselees when a variant is found but no screening is offered as it is unknown what this variant implies. Second, whether or not to test relatives when an unknown variant is detected.

(1) When finding a variant for the first time, you don’t know what the next steps should be… I feel uncomfortable telling that to counselees.

(2) Well, I feel particularly uncertain when something is found and it’s unclear what it implies for the family … will you offer to test the relatives? And what should you tell the family regarding risks? And will you give them advice about screening? I just don’t know. (FG 006)

Third, counsellors were uncertain about the course of action when no mutation is determined in families in whom a genetic predisposition for cancer is strongly suspected:

When no mutation is found, but we have the feeling there should be something, I wonder what it could be and what we should do next. (FG 002)

Counsellors indicated that the uncertainty about the extent of information provision i) reduces over time, when they realise that uncertain findings are relatively uncommon, and ii) also reduces when medical action can be offered after a variant has been identified:

i) It’s strange that it [feeling of uncertainty] becomes less. The more panel tests are carried out and nothing is found, the more I think that most cancers are not inherited.

Yes, that’s true … as not many uncertain variants are identified, I feel less uncertain about whether or not I have to inform counselees about that. I make that decision more easily. (FG 005)

ii) When you have a plan of action, you become less uncertain … even though you don’t know the exact meaning of a mutation. (FG 003)

Even though the feeling of uncertainty may reduce over time, most counsellors wanted more uniformity between their peers and between centres about the provision of information:

I’d like to have a national consensus, so that counsellors in each centre tell more-or-less the same. (FG 004)

During the interviews, counselees had difficulty expressing any uncertainties related to the panel tests, possibly due to impaired recall:

I have to say, I don’t remember it [uncertainty] clearly, so I think it wasn’t mentioned or only mentioned very briefly. What the panel test entails, what genes are tested and how, was explained clearly. But I think uncertainty about the test and what it entails was not mentioned.

(Counselee 11: a-61-year old women, cancer patient)

One form of uncertainty mentioned by many counselees was related to uncertainty regarding the detectability of a genetic cause due to limited knowledge in genetics. However, some stated that this uncertainty is fully understandable and did not trouble them during counselling:

They explained to me they will do everything possible. But they can’t guarantee that it isn’t hereditary when nothing is found. They don’t have the tools yet ... I find that understandable. With the current state of techniques and research, it’s only reasonable that some things are unknown.

(Counselee 5: a-65-year old man, cancer patient)

Nevertheless, other counselees wanted to reduce the uncertainty:

Well, I understand that this test doesn’t always result in an answer. However, if that is the case, I’ll go a step further until it actually provides certainty.

(Counselee 3: a-42-year old man, cancer patient)

### Discussing uncertainty

Counsellors varied in how much uncertain information they discussed with counselees during pre-test counselling; some provided more information on well-known mutations, whereas others focused more on unknown variants for which they cannot offer any screening to counselees:

Well, you talk about the genes that are well known … that’s what you focus on. That makes sense, because there’s more information to give about those genes.

Oh really? You don’t talk about uncertain variants? I specifically tell about the uncertain possible outcomes […], I think it’s important a counselee understands those. (FG 004)

Despite the observed variation in communication between counsellors, we found that some uncertain information was provided by almost all counsellors during pre-test counselling, i.e. information about i) the risk of developing cancer and inheriting a mutation, ii) about the possibility that no mutation will be identified, and iii) an example of an unknown variant that can be detected:

I think, well I hope, we all provide information about the specific cancers and their screening options, and the possibility that nothing is detected.

Also, I always emphasise some information about the possible test results. You know … a variant is detected that is clear to us, nothing is detected, and something unknown is detected,. (FG 002)

Many counsellors give a smaller amount of uncertain information to individuals with a lower level of education and to persons recently diagnosed with cancer:

I give less information to people with a low education level because it’s harder for them to understand. I try to structure the information, simply by using less text.

.. and the situation of the patient is also important. Was he recently diagnosed with cancer or has it been a while? That makes a difference about how much you can tell and what they understand. If someone has just been diagnosed, I give them less information … they are overloaded and not able to receive a lot of information. (FG 001)

Many counsellors provided more detailed information about uncertainty during post-test counselling than during pre-test counselling. They argued they did not want to overload the counselee with possibly irrelevant information:

During post-test counselling you give tailored advice for that specific counselee and their test result. For example, I go into detail about the possibility that we might know more about an unknown variant in a couple of years, only when such a variant is detected. I wouldn’t say that during pre-test counselling because I’ve already mentioned enough uncertainties. I don’t know if it will be relevant for this counselee. (FG 006)

Counsellors mentioned several strategies to help make the uncertain information as clear and understandable as possible for counselees, i.e. 1) giving general information about the genes to be tested, instead of providing details, 2) illustrating uncertain information by giving examples or using metaphors and (3) repeating information and providing a summary at the end of the consultation to aid the counselee’s recall of information:

(1) I will say ‘We’ll see whether we can find the hereditary cause. If we find a variant in one of those genes, we’ll look at the consequences’. I explain it very generally and do not specify what it entails for each gene. (FG 004)

(2) I sometimes use a book as a metaphor: ‘Multigene panel testing is comparable to reading a book. Some spelling mistakes are well known and some are new and unknown’… sometimes it helps people and sometimes it doesn’t.

(3) Yes, so do I. And most of the time I try to repeat information and provide a short summary of the three possible outcomes that they can expect. (FG 002)

Providing a summary during genetic counselling was considered important, and counselees expressed a need to receive printed information to take home:

It would have been good to receive a brief summary to know what was discussed in the consultation and what will be the next steps.

(Counselee 12: a-65-year old woman, cancer patient)

Lastly, counsellors always tried to be honest about uncertainty with their counselees. In turn, counselees hoped for and appreciated such openness:

I’d rather a doctor honestly tell me ‘I don’t know, I’m going to discuss this with my colleagues’ than just create some story.

(Counselee 3: a-42-year old man, cancer patient)

Furthermore, counselees experienced less uncertainty when their counsellor spoke calmly and understandably and provided enough space for questions:

She explained everything really clearly, also the things they don’t know yet. She used examples and also talked very calmly. I didn’t have many questions, but she provided enough room for questions. I felt comfortable. I don’t know, I just liked her.

(Counselee 7: a-48-year old woman, relative of multiple cancer patients)

### Decision making under uncertainty

Counsellors explained that, compared to targeted tests, discussing a multigene panel test during pre-test counselling results in a more explicit decision-making process due to the higher amount of uncertainty. Counsellors emphasised that counselees’ preferences are even more important because of the potentially harmful consequences of the test, such as having to deal with an uncertain test result:

In larger cancer panels that involve more uncertainty, I explain the test more extensively and the counselee’s opinion is much more important. I want counselees to really understand what they choose for.

Yes, I think the counselee should decide whether or not to do the test. If I make that decision and we detect an unknown mutation, they might end up confused because they can’t deal with it. (FG 005)

This corresponds with what most counselees experienced during their consultation:

It was important to me that I gave permission for testing, because it is a very informative test which can also be detrimental. So I was happy that I was asked whether I agreed - if I had said no, it would not have happened.

(Counselee 7: a-48-year old woman, relative of multiple cancer patients)

Counsellors sometimes advised counselees to postpone and reflect on their decision:

Regarding panel tests - I’m a bit cautious because I sometimes feel that people receive so much information. Since the test results can have serious consequences, even though the risks are low, I want to give people time to think about it. (FG 005)

However, counsellors thought that most counselees have clear preferences about whether or not they want to perform a panel test; comments of counselees were in line with this:

I actually didn’t need to think about it - I had already made the decision because I just wanted to know.

(Counselee 8: a-73-year old woman, cancer patient and relative of multiple cancer patients)

This readiness to decide made counsellors doubt whether counselees understood the uncertain information correctly, and whether they were fully aware of what they had chosen:

I find it difficult to judge whether someone is really able to give informed consent. You’re ‘only talking’ about seven additional breast cancer genes, and you’re not able to explain what happens if something else is detected. So, actually, they don’t know exactly what they’re agreeing with, but they will know whenever something [uncertain] is found. (FG 002)

Counselees indicated that uncertainty was not a reason for them to forgo testing:

Not performing a test because you might receive uncertain news - I don’t understand why you would do that.

(Counselee 4: a-58-year old woman, cancer patient and relative of cancer patient)

## Discussion and conclusion

### Discussion

This study is the first to explore how both counsellors and counselees experience and deal with uncertainty, and how decisions are made related to multigene panel testing for cancer, with the aim to further elucidate the advantages and drawbacks of panel testing (Howard and Iwarsson 2017).

The uncertainties that emerged, including well-known ones such as VUS and unsolicited findings (Han et al. 2017; Howard and Iwarsson 2017), are not unique to gene panels. However, the increased probability of detecting an uncertain test result when using these panels emphasises the importance of paying attention to uncertainties in genetic counselling (Bradbury et al. 2015; Kurian and Ford 2015). We showed that many uncertainties are discussed with patients by almost all counsellors, such as risks and examples of uncertain test results. However, the extent and manner of discussing uncertain information during genetic counselling were not always clear cut. As no guidelines exist, variation became apparent in our findings between how much uncertain information counsellors communicated and how. For example, some counsellors provide more information about uncertain variants, whereas others mainly outline information about well-known mutations. Our findings indicate that counsellors would like consensus regarding the provision of information on uncertainty during genetic counselling as well as a reduction in the variation in practice.

Counsellors tended to inform counselees more extensively after testing, which enabled them to tailor the information to the individual counselee and to avoid unnecessary anxiety during pre-test counselling. It is understandable that counsellors try to avoid confusing or even frightening counselees as previous studies have shown that awareness of uncertainty may increase patients’ anxiety, and can lead to decision avoidance and decision dissatisfaction (Politi et al. 2011; Politi et al. 2007). In the current study, counselees reported they were able to express their preferences during genetic counselling, and had a say in the decision about whether to perform a multigene panel. Nevertheless, it can be questioned whether this really was an informed choice. Considering that counsellors varied in the degree to which they discussed uncertainty during pre-test counselling, some counselees might have based their preferences and (subsequently) their decision on incomplete information. It is important to involve patients in decisions as it causes them to be more satisfied and enables them to weigh the pros and cons of multigene panel testing and make a more informed decision (Kunneman et al. 2016; Politi et al. 2011). Moreover, previous studies showed that patients better manage the perceived threat of uncertainty when they were aware of the possibility of receiving an uncertain test result (Politi and Street 2011; Rains and Tukachinsky 2015). Counselees were additionally found to appreciate receiving full and honest information, even when it encompassed uncertain information (Bijlsma et al. 2017). Not communicating uncertainty could be misconstrued as communication of certainty (Han 2013). Hence, not informing, and therefore not preparing counselees before testing, may increase the (psychological) impact of, for example, receiving an uncertain test result. However, a decision making process in which the patient participates entails more than providing information (Hargraves et al. 2016). It involves a doctor and a patient working together to make a decision that is in line with the patients’ situation and preferences (Lindor et al. 2016). Only presenting information, and maybe too much information, can cause patients to be overwhelmed and feel abandoned in the decision making process (Kunneman et al. 2016). Ideally, the content and approach for communicating uncertainty about multigene panel testing are standardised, and include information about potential uncertain test outcomes as well as an informed decision making process. This way undesired variation between counsellors whether and how they inform counselees is reduced as much as possible. One example is the informed consent model of Bradbury et al., which distinguishes what genetic information should be presented to all patients, and what information should be provided depending on patients’ needs (Bradbury et al. 2015).

In contrast to counsellors, counselees were less able to report uncertainties concerning multigene panel testing. Counselees in this study struggled to recall information, despite their relatively high educational levels, which is in line with previous findings (Culver et al. 2001; Geller et al. 1999; Glanz et al. 1999; Lerman et al. 1994). This could have two possible causes. First, recall may have been influenced by receiving the test result a relatively long time prior to the interview. Counselees suggested that because none of them had received uncertain or positive test results, their memory of which uncertainties had been discussed (before testing) might have been tainted. Second, other studies have shown that counselees mainly focus on receiving an answer to their genetic question during counselling and may not be open to receiving additional information (Michie et al. 1997; Shiloh et al. 2006). Counsellors should be aware of this when providing additional information to counselees, such as uncertainties. They may try to enhance counselees’ recall (e.g. of uncertainties) by slowly pacing the information.

### Strengths and limitations

One strength of this study is that its design, i.e. focus groups with counsellors and interviews with counselees, provided insight into the experiences of both groups and allowed to reflect on the similarities and differences between them. This provided a broad overview of the current way of experiencing and dealing with uncertainty related to multigene panel testing. Second, six of the eight Dutch centres providing genetic counselling participated, yielding a representative cross-section of the counsellors in the Netherlands. Additionally, the sample of counsellors varied in age, professional training, and years of experience. Finally, we interviewed counselees who were cancer patients, as well as counselees who had a relative with cancer.

A limitation is that, because we organised focus groups per centre, the discussions were limited to counsellors from within a single centre. However, we used quotations of previous focus groups to provoke responses from other centres. Second, to guarantee anonymity of the participants, we did not record who made which remark and could therefore not differentiate between the experiences of interns and residents, and experienced clinical geneticists. It would be interesting to investigate whether the experiences differ between job position and level of experience in future research. Third, only a small number of counselees could be interviewed, as multigene panels are not yet offered to all counselees. Lastly, due to practical reasons, the counsellors were responsible for informing counselees about the study. Although counsellors reported that all eligible counselees had been informed and only three declined, they might have selected counselees they preferred to be informed about this study and response rate may therefore differ.

### Conclusion

This study provides insight into counsellors’ and counselees’ experiences regarding uncertainty in cancer genetic counselling caused by multigene panel testing. Future research could focus on developing more uniformity in the extent and manner of providing appropriate information about uncertainty. Hopefully, this will contribute to improved communication in genetic counselling and enable better-informed decision making among counselees.

## Appendix

**Table 2:** Topic list for focus group sessions with counsellors

[Start and introduction of the focus group]	
1. Awareness of uncertainty and its consequences	
2. Counsellors’ experienced uncertainties	
3. Communication of uncertainty (using a scenario describing a counselee with multiple possible cancer syndromes visiting for cancer genetic counselling, and eligible for multigene panel testing)	
a. What uncertainties would you tell and what would you not tell this counselee?	
b. What are the reasons for (not) communicating uncertainties?	
4. Difficulties in communicating uncertainty	
[Break]	
5. Experienced uncertainties on the side of the counselee	
6. Decision making concerning multigene panel testing	
a. What are the difficulties?	
b. Whether and what is the influence of uncertainty in decision making?	
c. What are the differences in decision making when comparing multigene panel testing with targeted testing?	
7. Strategies to facilitate coping with uncertainty	
8. Counsellors’ needs regarding communicating uncertainty	
9. Post-test	
a. (What) are the remaining/created uncertainties?	
b. What are possible test results and counselees’ responses?	
c. Are there experiences with moments of regret?	
[Closure of the focus group]	

**Table 3:** Topic list for interviews with counselees

[Start and introduction of the interview]	
1. Counselee’s memory/recall of the genetic consultation	
a. What was the personal feeling/experience?	
b. What information (content) was provided?	
2. Uncertainty	
a. An explanation of uncertainty by the counselee.	
b. Optional: an additional explanation of uncertainty by the interviewer.	
c. What uncertain information is provided during genetic counselling? (When no uncertainty is recalled, examples of uncertain information are provided, such as ‘uncertainty about the meaning of a mutation’)	
3. Counselees’ personal experience of uncertainty	
[Break]	
4. Communication of uncertainty	
a. How was uncertainty communicated?	
b. How is this experienced?	
5. Decision making concerning multigene panel testing	
a. How was the decision regarding testing made?	
b. How is this experienced?	
c. What are the counselee’s preferences regarding decision making?	
6. Counselees’ needs regarding uncertainty	
[Closure of the interview]	
